# Trace elements can influence the physical properties of tooth enamel

**DOI:** 10.1186/2193-1801-2-499

**Published:** 2013-10-02

**Authors:** Elnaz Ghadimi, Hazem Eimar, Benedetto Marelli, Showan N Nazhat, Masoud Asgharian, Hojatollah Vali, Faleh Tamimi

**Affiliations:** Faculty of Dentistry, McGill University, Montreal, QC Canada; Department of Mining and Materials Engineering, McGill University, Montreal, QC Canada; Department of Mathematics and Statistics, McGill University, Montreal, QC Canada

**Keywords:** Crystal domain size, Trace elements, Tooth enamel, Physical properties

## Abstract

In previous studies, we showed that the size of apatite nanocrystals in tooth enamel can influence its physical properties. This important discovery raised a new question; which factors are regulating the size of these nanocrystals? Trace elements can affect crystallographic properties of synthetic apatite, therefore this study was designed to investigate how trace elements influence enamel’s crystallographic properties and ultimately its physical properties.

The concentration of trace elements in tooth enamel was determined for 38 extracted human teeth using inductively coupled plasma-optical emission spectroscopy (ICP-OES). The following trace elements were detected: Al, K, Mg, S, Na, Zn, Si, B, Co, Cr, Cu, Fe, Mn, Mo, Ni, Pb, Sb, Se and Ti. Simple and stepwise multiple regression was used to identify the correlations between trace elements concentration in enamel and its crystallographic structure, hardness, resistance to crack propagation, shade lightness and carbonate content. The presence of some trace elements in enamel was correlated with the size (Pb, Ti, Mn) and lattice parameters (Se, Cr, Ni) of apatite nanocrystals. Some trace elements such as Ti was significantly correlated with tooth crystallographic structure and consequently with hardness and shade lightness. We conclude that the presence of trace elements in enamel could influence its physical properties.

**Electronic supplementary material:**

The online version of this article (doi:10.1186/2193-1801-2-499) contains supplementary material, which is available to authorized users.

## Background

Tooth enamel is composed of both an organic and an inorganic phase. The organic phase is composed of proteins such as amelogenin, ameloblastin and tuftelin, as well as minor concentrations of proteoglycans and lipoids (Belcourt and Gillmeth 1979; Eggert et al. 1973; Glimcher et al. 1964). The enamel inorganic phase is composed of well-packed nanocrystals made of calcium phosphate apatite (HA) with small amounts of incorporated trace elements (Sprawson and Bury 1928). The organization and size of apatite crystals in tooth enamel affects its hardness (Jiang et al. 2005) and optical properties (Eimar et al. 2011,2012). These findings raise the following question: what determines the size of apatite crystals in tooth enamel? One possibility is that the tooth protein content could affect its crystal domain size, however we had found that the concentration of protein in enamel is not associated with the crystallographic structure of mature teeth (Eimar et al. 2012). Therefore this study was designed to investigate other factors, namely the presence of trace elements that can influence the size of apatite crystals in enamel.

The crystallographic properties of synthetic hydroxyapatite (HA) have been found to be influenced by the incorporation of trace elements (Table 1). Some of the trace elements expand the crystal cell lattice parameters of synthetic HA along the a-axis (Fe^2+^, Fe^3+^, Sr^2+^ and Zn^2+^ (molar fraction > 10%)) while others shrink it (SiO_4_^4-^, CO_3_^2-^, Mg^2+^, Zn^2+^ (molar fraction < 10%) and Ti^4+^). The crystal domain size along c-axis can be increased by some trace elements (SiO_4_^4-^, CO_3_^2-^, Zn^2+^, Fe^2+^, Fe^3+^and Sr^2+^) and decreased by others (Mg^2+^, Ni^2+^, Cr^3+^, Co^2+^ and Ti^4+^). Some trace elements can increase the crystallinity (degree of structural order of atoms) and crystal domain size (average length of individual crystals) of synthetic HA (Cr^3+^, Co^2+^ and Ni^2+^), while others have the opposite effect (SiO_4_^4-^, Zn^2+^, CO_3_^2-^, Fe^2+^, Ti^4+^, Sr^2+^, Ce^3+^ and Mg^2+^) (Christoffersen et al. 1997; Ergun 2008; Feng et al. 2005; Hu et al. 2007; Huang et al. 2011; Li et al. 2008; Lin et al. 2007; Mabilleau et al. 2010; Morrissey et al. 2005; Ren et al. 2010; Ribeiro et al. 2006; Tang et al. 2005; Wang et al. 2008).

**Table 1:** **Summary of the literature on the effect of trace elements on crystallography parameters in synthetic HA**

Crystallographic parameters	Trace elements that increase crystallographic parameters	Trace elements that decrease crystallographic parameters
Lattice along a-axis	Fe^2+^, Fe^3+^, Zn^2+a^, Sr^2+^	SiO_4_^4-^, CO_3_^2-^, Zn^2+ b^, Ti^4+^
Lattice along c-axis	Fe^2+^, SiO_4_^4-^, CO_3_^2-^, Zn^2+^, Fe^3+^, Sr^2+^	Mg^2+^, Ti^4+^, Co^2+^, Ni^2+^, Cr^3+^
Crystallinity	Co^2+^, Ni^2+^, Cr^3+^	SiO_4_^4-^, CO_3_^2-^, Zn^2+^, Fe^3+^, Ti^4+^, Mg^2+^, Ce^3+^
Crystal domain size along c-axis	Co^2+^, Ni^2+^, Cr^3+^	Fe^2+^, SiO_4_^4-^, CO_3_^2-^, Zn^2+^, Ti^4+^, Mg^2+^, Ce^3+^

^a^ molar fraction >10%; ^b^ molar fraction < 10%.

Unlike the effect of trace elements on synthetic HA, their role on crystallographic properties of enamel is unknown in the literature. Despite the very low concentration of trace elements in our body, they play a significant role in human body healthiness (Carvalho et al. 1998). Trace elements can enter our body through digestion of food or by exposure to the environment (Lane and Peach 1997) and they can be incorporated into the structure of enamel HA. Trace elements in tooth enamel have been investigated for their role in caries (Curzon and Crocker 1978) and it was found that the presence of F, Al, Fe, Se and Sr is associated with the low risk of tooth caries, while Mn, Cu and Cd have been associated with a high risk (Curzon and Crocker 1978). However, despite their apparent importance on tooth enamel homeostasis, the effect of trace elements on the crystallography and physical properties of enamel remains unknown.

The aim of this study is to find the correlation between the concentration of trace elements detected in tooth enamel and its crystallography and physical-chemical properties. We hypothesized that the incorporation of trace elements in the structure of enamel can affect its crystallography and consequently alter the physical properties of enamel.

## Results

### Physical chemical properties of tooth enamel

**Figure 1:**
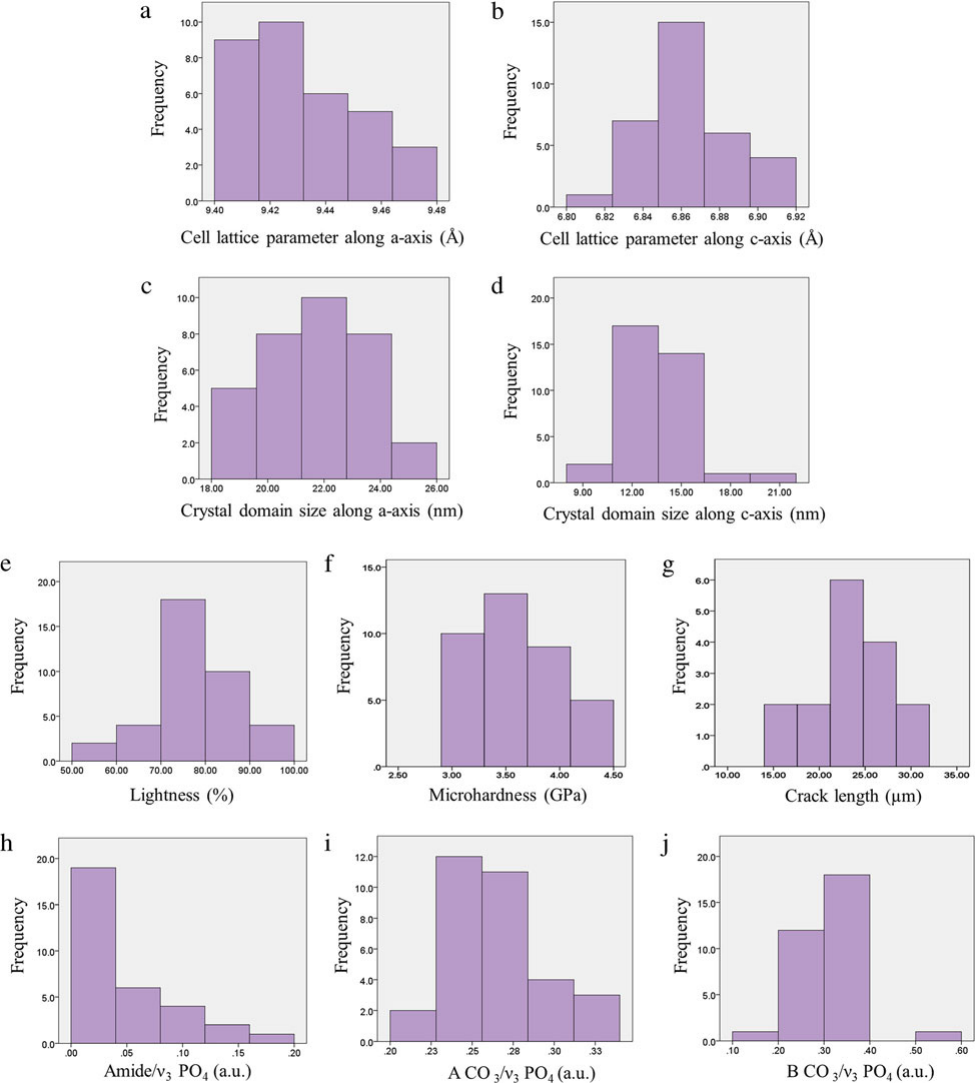
**Frequency histograms describing the variations in physical-chemical properties of tooth enamel:** cell lattice parameters along **(a)** a-axis and **(b)** c-axis; crystal domain length along **(c)** a-axis and **(d)** c-axis; **(e)** tooth shade lightness, **(f)** hardness and **(g)** average crack length; **(h)** the organic relative content and carbonate relative content [**(i)** type A and **(j)** type B] in enamel mineral matrix among the examined teeth.

Among all tooth enamel samples, cell lattice parameters varied along a-axis between 9.40 and 9.47 Å (mean = 9.43 ± 0.004 Å) and along c-axis between 6.84 and 6.92 Å (mean = 6.86 ± 0.004 Å). Unlike the cell lattice parameter along c-axis, the cell lattice parameter along a-axis followed a normal distribution among all samples (Figure 1a, 1b). Crystal domain size along a-axis ranged between 10.31 and 18.08 nm (mean = 13.49 ± 0.349 nm) and along c-axis varied between 18.09 and 25.85 nm (mean = 21.7 ± 0.33 nm) following a normal distribution (Figure 1c, 1d).

The tooth enamel hardness, crack length and shade lightness followed a normal distribution among the samples analyzed (Figure 1e, 1f and 1g). The hardness values varied between 2.91 and 4.36 GPa (mean = 3.64 ± 0.08 GPa), the crack length varied between 14.60 and 30.02 μm (mean = 23.31 ± 1.49 μm) and the tooth shade lightness values ranged between 59.0 and 96.1% (mean = 79.0 ± 1.8). The relative content of apatite inorganic carbonate type A and type B among the enamel samples followed a normal distribution, while the relative organic content did not (Figure 1h, 1i, 1j).

### Trace elements in tooth enamel

**Figure 2:**
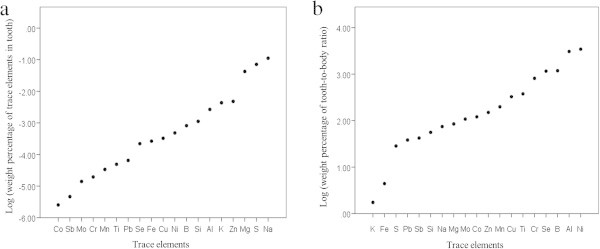
**The concentration of trace elements.** The figures show **(a)** the logarithm of trace elements concentration in tooth enamel and **(b)** the logarithm of tooth-to-body concentration ratio.

A total of 19 trace elements were detected in the tooth enamel samples by ICP (see Additional file 1: Table S1). The concentration of the different trace elements varied considerably among tooth enamel samples (Figure 2a). Cr, Mo, Co and Sb had the lowest concentration in tooth enamel compared with other trace elements while Zn, Na and S had the highest one. In order to assess how concentrated were the trace elements in enamel compared to the rest of the body, they were normalized to the average elemental composition of the human body (Frieden 1972; Glover 2003; Zumdahl and Zumdahl 2000). Among the 19 trace elements detected, some of them had a similar concentration in tooth enamel compared to the rest of the body (K and Fe), while others were concentrated by 1 (S, Sb, Pb, Si, Na and Mg), 2 (Mo, Co, Zn, Mn, Cu, Ti and Cr), or 3 orders of magnitude (Se, B, Al and Ni). The largest difference in concentration of trace elements between tooth enamel and the rest of the body belongs to Ni, which Ni is close to 3500 times more abundant in tooth enamel than in the rest of the body.

### The correlation analyses of trace elements

**Figure 3:**
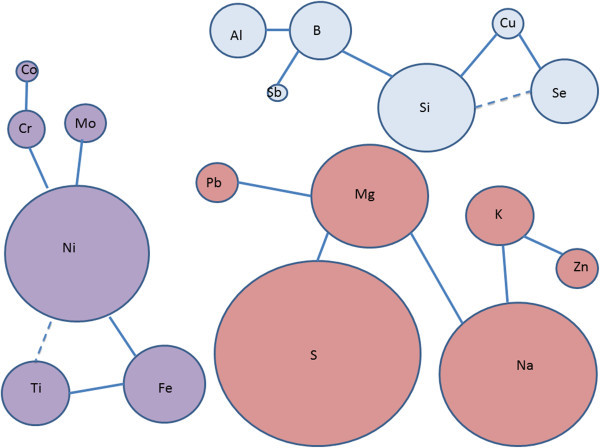
**The correlation between trace elements in tooth enamel.** In scheme straight lines represent the positive correlations and dotted lines represent the negative ones. Differences in the size of circles are directly proportional to the concentration difference among the correlated elements.

The simple linear regression was used to find the correlation between each possible pair combination of trace elements in tooth enamel (see Additional file 1: Table S2). It was found that the following correlations between trace elements were significant: Al-to-B, Al-to-Sb, Al-to-Si, B-to-Sb, B-to-Si, B-to-Cu, Co-to-Cr, Cr-to-Cu, Cr-to-Ni, Cr-to-Si, Cu-to-Sb, Cu-to-Se, Cu-to-Si, Fe-to-K, Fe-to-Na, Fe-to-Ni, Fe-to-S, Fe-to-Ti, Fe-to-Zn, K-to-Mg, K-to-Mo, K-to-Na, K-to-Ni, K-to-Pb, K-to-S, K-to-Zn, Mo-to-Ni, Na-to-Ni, Na-to-Pb, Na-to-S, Na-to-Zn, Ni-to-S, Pb-to-S, S-to-Zn, Sb-to-Si and Ti-to-Zn (see Additional file 1: Table S2). Stepwise multiple regression was done to find the correlation between pairs combination of trace elements adjusting for the presence of other trace elements in tooth enamel. The significant correlations among trace elements concentration using stepwise multiple regression were: Al-to-B, Al-to-Si, B-to-Si, Cu-to-Si, K-to-Mg, K-to-Na, B-to-Cu, Mg-to-Na, Na-to-S, S-to-K, S-to-Mg. The findings showed three independent groups of elements that were directly or indirectly correlated to each other (Figure 3). In the first group, there was a positive correlation among Al-to-B, B-to-Si, B-to-Sb, Si-to-Cu, Cu-to-Se, and a negative correlation between Si and Se. In the second group, Co-to-Cr, Cr-to-Ni, Ni-to-Fe, Fe-to-Ti and Ni-to-Mo were correlated positively; while Ni-to-Ti were correlated negatively. In the third group, all of the correlations (Pb-to-Mg, Mg-to-Na, Na-to-K, K-to-Zn and Na-to-S) were positive. Mn was the only element that was not correlated to any other element.

### Simple linear regression between the concentration of elements and tooth properties

The correlations between trace element concentration and physical-chemical parameters (crystallographic parameters, hardness, crack length, shade lightness and carbonate content) of enamel samples are presented in Additional file 1: Table S3 and the significant ones are summarized in Table 2. There was a strong positive correlation between the concentration of Ti in enamel and tooth hardness, lightness and apatite crystal domain size along c-axis. The correlations between Fe concentration in enamel and both tooth lightness and carbonate type A content in tooth enamel were significant. Also, the correlations between Cu concentration in enamel and both crystal domain size and cell lattice parameter along a-axis were significant. There was a negative association between Al concentration and tooth enamel crack length. A strong inverse relationship was observed between the concentration of Pb and crystal domain size along c-axis. The correlations between the concentration of Se and cell lattice parameter along both a-axis and c-axis were significant. A negative strong correlation was seen between the concentration of Cr and cell lattice parameter along c-axis. Also, there were significant correlations between the concentration of Ni and both, cell lattice parameter along c-axis and carbonate type B. The association between the concentration of S and carbonate type B was significant. The remaining chemical and crystallographic parameters were not correlated to each other.

**Table 2:** **Simple linear and stepwise multiple correlation between trace elements’ concentration in enamel and tooth properties**

Correlated properties	Correlated elements	Simple linear regression			Multiple stepwise regression		
		R	B	P	R	B	P
Mechanical properties							
Hardness	Ti	0.34	27.00	0.037	0.34	2753.54	0.037
Average crack length	Al	0.67	−18.08	0.005	0.70	−18.08	0.005
Optical properties							
Lightness	Fe	0.46	153.60	0.004	NS	NS	NS
˶	Ti	0.47	832.79	0.003	0.47	832.79	0.003
Crystallographic structure							
Crystal domain size along a-axis	Cu	0.40	32.87	0.019	NS	NS	NS
Crystal domain size along c-axis	Pb	0.52	−199.11	0.002	0.70	−138.76	0.019
˶	Ti	0.51	−192.05	0.002	˶	−133.19	0.02
˶	Mn	NS	NS	NS	˶	184.01	0.025
Cell lattice parameter along a-axis	Cu	0.35	0.24	0.045	NS	NS	NS
˶	Se	0.59	0.36	<0.001	0.59	0.36	<0.001
Cell lattice parameter along c-axis	Cr	0.38	−6.97	0.031	0.72	−5.79	0.031
˶	Ni	0.40	−0.09	0.02	˶	−0.06	0.047
˶	Se	0.52	0.35	0.002	˶	0.36	<0.001
Carbonate Content							
Type A	Fe	0.41	−0.61	0.021	0.41	−0.61	0.021
Type B	Co	0.37	−18.44	0.007	0.77	−16.15	0.011
˶	Ni	0.70	0.47	<0.001	˶	0.46	<0.001
˶	S	0.43	0.01	0.014	NS	NS	NS

R: the correlation coefficient; B: the regression coefficient; P: the significance of Pearson correlation and NS: not significant.

### Stepwise multiple linear regression between elements and tooth properties

The results of stepwise multiple regression between the concentration of trace elements in enamel and the mechanical properties, optical properies, crystallographic structure and carbonate content of the teeth are presented in Table 2. Most of the results from the stepwise multiple regression analysis confirmed the results of the simple linear regression. It was found that the concentration of Ti was associated with tooth enamel hardness, lightness and crystal domain size along c-axis. The concentration of Pb and Ti in enamel had a negative correlation with tooth enamel crystal domain size while Mn had a positive one. The correlation between the concentration of Al and crack length in tooth enamel was negative. The concentration of Se was associated positively with the cell lattice parameters along both a-axis and c-axis. The concentration of Cr and Ni had an inverse correlation with the cell lattice parameter along c-axis. The concentrations of Fe and Co were negatively correlated to the carbonate type A and type B, respectively. The concentration of Ni and carbonate type B were associated positively. The protein content in tooth enamel was correlated to none of the trace elements. Some of the trace elements such as Fe, S and Cu were correlated to the enamel properties by stepwise multiple regression but not by simple linear regression and vice versa (such as Mn).

## Discussion

In this study 12 trace elements were found to be associated with tooth composition, structure and physical properties. Underneath we compared our findings with previous studies and we discussed the possible sources of these contaminants.

### Trace elements in tooth enamel

Trace elements enter the human body coming from different sources such as food, water, air, etc. (Kampa and Castanas 2008; Malczewska‒Toth 2012). They can be incorporated in tooth enamel structure and in this study we show that they could affect the physical chemical properties of enamel. Below the association between each detected trace elements in tooth enamel with the tooth physical and chemical properties are detailed.

### Selenium

Selenium is a non-metallic element widely distributed in nature that can be absorbed by the body through oral intake or breathing (Malczewska‒Toth 2012). Selenium is incorporated in synthetic HA through anionic exchange of phosphate with selenite in a one-to-one (1:1) substitution ratio (Monteil-Monteil-Rivera et al. 2000). In our study, we found that the presence of Se in enamel was associated with increased lattice parameters along a-axis and c-axis. This was logically expected because the ionic radius of Se^4+^ (0.50 Å) is larger than the ionic radius of P^5+^ (0.35 Å), so by substitution of Se in synthetic HA, the lattice parameters increase (Ma et al. 2013). However ours is the first study that reports this phenomenon in tooth enamel.

### Chromium

Chromium is a heavy metal that is essential for the body in small amounts (Kampa and Castanas 2008). Its main role is in controlling the fat and sugar metabolism (Kimura 1996), and it helps to increase the muscle and tissue growth (Schroeder et al. 1963). It can enter human body through water, air and food (Kampa and Castanas 2008).

Although the ionic radius of Ca^2+^ (0.99 Å) is much bigger than Cr^3+^ (0.69 Å), Cr can exchange with Ca in synthetic HA (Chantawong et al. 2003). Accordingly, the substitution of Cr^3+^ in synthetic HA decreases the cell lattice parameters along both a-axis and c-axis, which might explain why we observed a significant association between the concentration of Cr and cell lattice parameter along c-axis in tooth enamel (Mabilleau et al. 2010).

### Nickel

Nickel is a toxic metal that can be absorbed by the body through water, air or food (Kampa and Castanas 2008). Ni is incorporated in HA through substitution of Ca^2+^(I) and bonds with O to form Ni_3_PO_4_ (Zhang et al. 2010). The ionic radius of Ca^2+^ (0.99 Å) is much bigger than Ni^2+^ (0.72 Å) (Chantawong et al. 2003). Consequently, with addition of Ni^2+^, the cell lattice parameter along c-axis decreases in synthetic HA (Mabilleau et al. 2010). These observations are in agreement with our study which to best of our knowledge is the first to report the inverse association between the concentration of Ni and crystal domain size in tooth enamel. Also, we found that the substitution of Ni in tooth enamel had a strong positive association with the presence of carbonate type B. Future studies will have to be performed in order to understand this phenomenon.

### Cobalt

Cobalt is a toxic metal that exists in the environment and can enter the body through water, air and food (Barceloux and Barceloux 1999; Duruibe et al. 2007). In HA, Ca^2+^ is substituted by Co^2+^ following the equation Ca_10-x_ Co_x_ (PO_4_)_6_ (OH)_2_ (Elkabouss et al. 2004) and the maximum exchange of Co with Ca is 1.35 wt% Co (Elkabouss et al. 2004). In this study, we report for the first time that the incorporation of Co in tooth enamel structure had a strong negative association with the substitution of carbonate type B. More studies are required to find the reason behind this phenomenon.

### Lead

Lead is a poisonous heavy metal that can harm the human body (Shukla and Singhal 1984). It can enter the body through water, air or food (Kampa and Castanas 2008). In HA, at low concentrations, Pb^2+^ ions (1.2 Å) replace Ca^2+^ ions (0.99 Å) (Mavropoulos et al. 2002; Miyake et al. 1986; Prasad et al. 2001) at the calcium site II following the equation Pb_(10-x)_Ca_x_(PO_4_)_6_(OH)_2_ (Mavropoulos et al. 2002). Accordingly the sorption of low concentrations of lead by synthetic HA decreases its crystal domain size (Mavropoulos et al. 2002) and this could be the reason why we found in our study that the presence of lead had a negative correlation with the size of enamel apatite crystal.

### Titanium

Titanium is a metal commonly used in the field of biomaterials and bio-applications (Niinomi 2002). Titanium characteristics such as high strength, low modulus of elasticity, low density, biocompatibility, complete inertness to body environment and high capacity to integrate with bone and other tissues make it widely used in implant applications (Niinomi 2002). Ti ions are absorbed by body through food like candies, sweets and chewing gums (Weir et al. 2012).

The ionic radius of Ti^4+^ (0.68 Å) is much smaller than the ionic radius of Ca^2+^ (0.99 Å) (Ribeiro et al. 2006), so the substitution of Ca by low concentrations of Ti in synthetic HA results in decreased cell lattice parameters and crystal domain size (Ergun 2008). We found that the concentration of Ti in tooth enamel HA was associated with decreased enamel crystal domain size, which is in agreement with the previous studies on synthetic HA (Ergun 2008; Hu et al. 2007; Huang et al. 2010).

In this study, the presence of Ti in tooth enamel was associated with increasing tooth hardness. Since Ti had an inverse association with the crystal domain size and the size of apatite nanocrystals in tooth enamel is inversely correlated to tooth hardness (Eimar et al. 2012), this could be the reason behind the positive association between Ti concentration in enamel apatite and hardness.

We previously showed that the tooth enamel crystal domain size was associated with its optical properties; when the enamel crystal domain size is larger, its lightness is lower (Eimar et al. 2011). This phenomenon is due to the fact that more light can be scattered from tooth enamel composed of small crystals (Eimar et al. 2011). In this study we found that the presence of Ti in tooth enamel structure was associated with both smaller crystal domain size and higher lightness, which confirmed our previous observation (Eimar et al. 2011).

### Manganese

Manganese is another trace element that can be uptaken from food, air and water (Frieden 1984; Kampa and Castanas 2008). Mn^2+^ replaces Ca^2+^ in HA (Medvecky et al. 2006). Previous studies have shown that the incorporation of Mn^2+^ in synthetic HA does not change the crystal domain size significantly (Medvecky et al. 2006; Ramesh et al. 2007). In our study we showed that the presence of Mn in tooth enamel was associated with its apatite crystal domain size. Further studies are needed to understand this phenomenon.

### Iron

Iron is an essential element for human life, it can enter the body through food such as vegetables and it is one of the trace elements found in teeth (Cook et al. 1972). Fe affects the carbonate content in synthetic HA (Low et al. 2010). It was found that in low concentration of Fe, Carbonate type A can be substituted by Fe in synthetic HA (Low et al. 2010). We found that the incorporation of Fe in tooth enamel had lower relative content of carbonate type A. Further studies are needed to understand this phenomenon.

### Aluminum

Aluminum is one of the elements found in the body that can be absorbed through air, food, and water (Campbell et al. 1957; Maienthal and Taylor 1968; Oke 1964). Its concentration in the body increases with age and at higher amounts can cause brain and skeleton disorders (Alfrey et al. 1976; Little and Steadman 1966). Also, the discoloration of tooth enamel can be seen in the presence of Al (Little and Steadman 1966). In this study, we found that the concentration of Al was correlated negatively with crack length in tooth enamel. Teeth with lower level of Al were more prone to have longer cracks and vice versa. More studies are needed to understand this phenomenon.

### Sources of trace elements

Trace elements are distributed differently among tooth enamel and dentin. For example Cu, Pb, Co, Al, I, Sr, Se, Ni and Mn are more abundant in tooth enamel compared to dentine while Fe and F are more concentrated in dentine than enamel (Derise and Ritchey 1974; Lappalainen and Knuuttila 1981). Also trace elements vary among the different layers of enamel. Fe, Pb and Mn are more abundant in the outer layers of enamel compared to the inner ones (Brudevold and Steadman 1956; Reitznerová et al. 2000). These findings seem to indicate that certain trace elements could be coming from the environment (i.e. Mn and Fe) and are incorporated after eruption or deposed in tooth enamel during calcification (Brudevold et al. 1975; Nixon et al. 1966; Okazaki et al. 1986). Trace elements can enter the human body coming from different sources such as food, water and air (Cleymaet et al. 1991; Cook et al. 1972; Duruibe et al. 2007). Underneath we discuss the dental products and fluids (i.e. saliva, dental prosthesis and dental porcelain) as possible sources of trace elements in tooth enamel.

### Saliva

Teeth are washed constantly by saliva (Duggal et al. 1991). Several trace elements found in saliva are known to affect the composition of enamel surface (Reitznerová et al. 2000). The concentration of trace elements in saliva varies among people. These are some of the elemental detection in enamel with the range of Cu (20–321 μgL^-1^), Zn (28–358 μgL^−1^), Mg (2.53-34.13 mgL^-1^), Ca (21.5-170 mgL^-1^), Al (25–102 μgL^−1^), Sr (9–26 μgL^−1^), Mn (2.3-5 μgL^−1^), Fe (25–160 μgL^−1^), Si (2–3 μgL^−1^), Na (257.49-276.8 mg/L), K (958.73-994.704 mg/L) and Cr (0.8-3.6 μgL^−1^) (Borella et al. 1994; Grad 1954; Sighinolfi et al. 1989).

The most abundant trace elements in saliva (Na, Mg, K and Zn) are also the most abundant trace elements in tooth enamel (Borella et al. 1994; Grad 1954; Sighinolfi et al. 1989). Interestingly, in our study the concentration of each one of these trace elements was directly or indirectly correlated to each other. These observations indicated that saliva could influence the composition of enamel.

### Dental prosthesis

Partial denture could be a possible source of trace elements found in teeth. The alloys commonly used to fabricate dental prosthesis include Cr, Co, Ni, Fe, Ti and Mo (Andersson et al. 1989; Asgar et al. 1970; Morris et al. 1979; Yamauchi et al. 1988). The concentration of Cr, Co, Fe and Ni in saliva of patients with partial dentures is higher than in patients without partial dentures (de-Melo et al. 1983; Gjerdet et al. 1991).

The concentration of Cr, Co, Mo, Ni, Ti and Fe in tooth enamel was correlated strongly to each other (Figure 3). This finding along with the fact that these materials are found in the composition of dental prosthesis seems to indicate that the source of these metals in enamel could be dental prosthesis. Therefore, the presence of denture in mouth can affect the concentration of trace elements in tooth enamel. Future studies will be performed to confirm the effect of trace elements found in dental prosthesis in tooth enamel.

### Dental porcelain

Dental porcelain is composed of a leucite crystallite phase and a glass matrix phase (Panzera and Kaiser 1999) and its elemental composition includes Si (57-66%), B (15-25%), Al (7-15%), Na (7-12%), K (7-15%) and Li (0.5-3%) (Panzera and Kaiser 1999; Sekino et al. 2001). Interestingly, the concentration of these elements in tooth enamel was strongly correlated to each other (Figure 3). This result seems to indicate that the dental porcelain might be another possible source of these elements in tooth enamel. Although future studies will have to be performed to confirm this possibility.

### Clinical implications

Trace elements can enter the structure of tooth enamel and affect its physical-chemical properties. In this study, we found that there are several sources for trace elements to enter enamel structure such as saliva, dental prosthesis or dental porcelain. Future studies will have to be performed to determine the effect of saliva and dental prosthesis on tooth enamel structure.

Metallic components of dental prosthesis are usually based on Cr, Co and Ni. These metals can cause sensitivities and allergies (Blanco-Dalmau et al. 1984; Brendlinger and Tarsitano 1970) and for these reasons Ti based dentures have been developed (Andersson et al. 1989; Yamauchi et al. 1988). In our study, we confirmed that the presence of Ti in tooth enamel could be beneficial by rendering teeth whiter and harder. Therefore, dental materials containing Ti could have additional benefits, besides the ones that already are known and used in biomaterial sciences.

### Limitations

One limitation of the present study is relatively small sample size and another one is the limited number of detected trace elements in each tooth sample. With increasing the number of sample size or using another technique in order to promote the detection limit, we might find more trace elements in the samples and more significant correlations between the concentration of trace elements and the physical-chemical properties of it.

## Conclusion

The presence of trace elements in tooth enamel could influence the physical chemical properties of tooth enamel. In this study we found that the concentration of Ti in tooth enamel had a strong relationship with enamel hardness, lightness and crystal domain size along c-axis. The incorporation of Fe had a negative association with the presence of carbonate type A, while the incorporation of Co and Ni was correlated with the formation of carbonate type B. The concentration of Al in tooth enamel was inversely correlated with the length of cracks forming in enamel. Also, we found that the presence of Se in tooth enamel had a positive correlation with cell lattice parameters along both a-axis and c-axis while Cr and Ni had a negative correlation with cell lattice parameters along c-axis. Also, it was shown that the concentration of Pb and Mn in tooth enamel had a positive association with tooth enamel crystal domain size along c-axis.

## Materials and methods

After obtaining ethical approval from McGill University Health Center (MUHC) ethical committee, a set of 38 extracted human teeth were collected from patients attending McGill Undergraduate Dental Clinic, scheduled for extractions. In this study, the included teeth were sound human upper anterior teeth. Teeth with caries, demineralized areas, cracks, cavitations, restorations, severe or atypical intrinsic stains, and/or tooth bleaching history were excluded.

Upon extraction, teeth were immersed in 10% formalin solution (BF-FORM, Fisher Scientific, Montreal, Canada) for 1 week, before cleaning them in an ultrasound bath (FS20D Ultrasonic, Fisher Scientific, Montreal, Canada) with de-ionized distilled water at 25°C for 60 minutes. Then, they were polished with a low-speed dental handpiece (M5Pa, KAB-Dental, Sterling Heights, MI) using SiC cups (Pro-Cup, sdsKerr, Orange, CA) and dental prophylaxis pumice of low abrasive capability (CPR™, ICCARE, Irvine, CA) for 1 minute. Teeth were rinsed again in an ultrasonic bath with de-ionized distilled water before storing them in labeled Eppendorf tubes with a 10% formalin solution.

### Tooth spectrophotometry

Tooth shade was registered by tooth spectrophotometry (Easy shade®, Vita Zahnfabrik, Germany) which is the most accurate and reproducible technique used for tooth shade masurements (Chu et al. 2010; Paul et al. 2002). Shade measurements were collected using the parameters of Munsell’s colour system (L*C*H*) and they were repeated three times for each tooth. The mean and the standard deviation for each shade parameter were calculated. Tooth dehydration might induce changes in tooth shade so, in order to avoid its dehydration, during shade measurement each tooth was kept wet at all times.

### Vickers microhardness

A sagittal section was obtained from each tooth using a carbide bur (FG56, sds Kerr, Orange, CA) adapted to a high-speed dental handpiece (TA-98LW, Synea, Bürmoos, Austria) and cooled with de-ionized distilled water in order to prevent overheating. Each tooth section was fixed in clear methylmethacrylate resin (DP-Ortho-F, DenPlus, Montreal, QC). The resulting blocks were mirror polished using ascending grits of silicon carbide papers with de-ionized distilled water (Paper-c wt, AAAbrasives, Philadelphia, PA) (240, 400, 600, 800 and 1200) and were smoothed with a polishing cloth.

A Vickers microhardness device (Clark CM100 AT, HT-CM-95605, Shawnee Mission, KS) was used to make indentations on the polished surfaces of tooth enamel. The indentation load was 300 N with a loading time of 10 seconds. Due to the variation in microhardness values within each tooth enamel sample, six indentations were performed between the DEJ and external surface of each enamel sample (Bembey et al. 2005; Gutiérrez-Salazar and Reyes-Gasga 2003; Newbrun and Pigman 1960; White et al. 2000). A minimum distance of 50μm was maintained, between the successive indentations. A computer software (Clemex Vision PE 3.5, Clemex Technologies Inc, Shawnee Mission, KS) was used to measure the microhardness value at the site of indentation from images captured with a built-in camera.

### Enamel crack propagation

**Figure 4:**
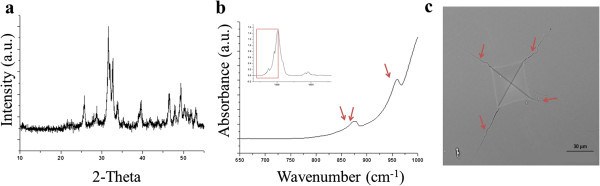
**XRD, FTIR spectrums and SEM micrograph of Vicker’s microindentation on tooth enamel samples. (a)** XRD spectrum of tooth enamel powder, **(b)** FTIR absorbance spectra of tooth enamel samples normalized to absorbance peak of ν_3_PO_4_ at 1013 cm^-1^(arrows show the peaks that used for the calculation of carbonate content), **(c)** SEM micrograph of Vicker’s microindentation on enamel (cracks are shown by arrows on the corners of indentation).

Indentations were made on the polished surfaces of tooth enamel that prepared as described above, with a Vickers microhardness device (Clark CM100 AT, HT-CM-95605, Shawnee Mission, KS). Half way between the DEJ and the surface of enamel 7 indentations were applied on each tooth enamel sample. Between the successive indentations, a minimum distance of 250μm was maintained. Indentation load was 500N with a loading time of 10 seconds. Upon indentation, cracks emanated from the corners of each indentation. Then, the samples were sputter-coated with gold and images of the cracks were captured with a VP-SEM (Hitachi S -3000N VP, Japan) at 500 magnification (Figure 4c). The length of the cracks was measured using the ImageJ software (US National Institutes of Health, Bethesda, MD). The average crack length for each indentation was calculated by summing up the length of cracks and dividing by the number of cracks (Chicot et al. 2009; Roman et al. 2002).

### X-ray powder diffraction

To determine the crystallographic dimension of apatite in each tooth enamel sample, X-ray powder diffraction (XRD) (D8-Discover/GADDS, Bruker, Karlsruhe, Germany) was used. The XRD patterns were recorded using a diffractometer with CuK_α_ radiation (setting: 40 Kv, 40 mA, 10-60° scanning angle, 0.02 step size and 1800 scan step time) (Hanlie et al. 2006; Xue et al. 2008). DIFFRAC-plus EVA software (AXS, Bruker, Karlsruhe, Germany) was used to analyze the data obtained from each XRD spectrum (Figure 4a) (Xue et al. 2008).

The average HA crystal domain size along c-axis and a-axis for each enamel sample were calculated using the (002) and (310) Bragg peaks of the XRD spectrum and Scherrer’s formula (Eq. 1)1

where D is the average of domain lengths, K is the shape factor, λ is the x-ray wavelength β is the line broadening at half the maximum intensity (FWHM) and θ is the Bragg angle.

The enamel crystal cell lattice parameters, a-axis and c-axis were calculated using the XRD (002) and (310) Bragg peaks relying on the following equation (Hanlie et al. 2006),2

where d is the spacing between adjacent planes (interplanar spacing) in the crystal, hkl are the miller indices that are the reciprocal intercepts of the plane on the unit cell axes, a is the a-axis and c is the c-axis. We used the (002) and (310) Bragg peaks for our crystallography calculation, because they have been widely used in the literature and they do not overlap with other peaks (Hanlie et al. 2006; Leventouri et al. 2009; Simmons et al. 2011).

### FTIR

The chemical composition of enamel was investigated by FTIR spectroscopy (Spectrum 400, Perkin–Elmer, Waltham, MA). The enamel powder samples were submitted to an FTIR spectrophotometer using a single bounce ZnSe diamond-coated ATR crystal. For each sample, a total of 64 scans per run at 2 cm^-1^ resolution were used (Figure 4b). FTIR studies were carried out in the range 700–1800 cm^-1^. The collected spectra were normalized according to the absorbance of ν_3_PO_4_ at 1013cm^-1^ using the FTIR spectrophotometry software (Spectrum, Perkin-Elmer, USA).

According to previous studies, the organic content of enamel was estimated from the Amide I-to-ν_3_PO_4_ ratio (Aparicio et al. 2002; Bartlett et al. 2004; Bohic et al. 2000). The carbonate content within enamel mineral matrix was estimated from the ratios of ν_2_CO_3_ type A (~878cm^-1^) and B (~872cm^-1^) to the ν_3_PO_4_ and ν_1_PO_4_ (~960cm^-1^) absorption bands (Antonakos et al. 2007; Lasch et al. 2002; Rey et al. 1989).

### Inductively coupled plasma-optical emission spectroscopy

The concentration of trace elements in tooth enamel samples was determined with Inductively Coupled Plasma-Optical Emission Spectroscopy (ICP-OES) (Thermo Scientific iCAP 6500, Cambridge, UK). Weighed tooth enamel powdered samples were dissolved in concentrated nitric acid (5 ml; 68% wt/wt) at a temperature of 95°. Yttrium (5 ppm) was added to the solution as an internal standard to make corrections for possible sample preparation errors and sample matrix corrections. After 2 hours of acid digestion, the liquids of the resulting solutions (0.25 ml) were diluted into both deionized-distilled water (10 ml) and 4% nitric acid (25 ml), separately. Both diluted solutions were submitted to ICP-OES using the following setup: power of 1150W, auxiliary gas-flow rate of 0.5 L/min, nebulizer gas-flow rate of 0.5 L/min, sample flow rate of 0.7 ml/min, cooling gas of 12 L/min and integration time of 10 sec. The operating software ITEVA (version 8) was used to control the instrument function and data handling. The quality control checks were done prior to initial analysis and every 12 consecutive samples.

### Data analysis

The correlations between the concentration of each trace element in tooth enamel and its physical chemical properties were determined using simple linear regression. Due to high correlations between trace elements in tooth enamel and their possible effect on the results, in addition to simple linear regression, stepwise multiple regression was performed. Stepwise multiple regression is more accurate than simple linear regression, because it provides us information about the correlation of each trace element with others while adjusting for their inter-correlations. Also, the association of trace elements with physical-chemical properties of tooth enamel were obtained using the stepwise multiple regression, adjusting for the inter-correlation between trace elements. The statistical significance was set at P <0.05 and all statistical analyses were done using SPSS 19 software (IBM, New York, NY).

## Electronic supplementary material

Additional file 1: Table S1: The frequencies and concentrations of 19 trace elements found in 38 human tooth enamel samples analyzed in this study. **Table S2.** The simple linear regression among trace elements in tooth enamel. The numbers in the table represent the regression coefficient and the color demonstrates the magnitude of the correlation (see legend). **Table S3.** Simple linear regression between elements and tooth properties. (DOCX 47 KB)
