# Supplementary material for: Trace elements can influence the physical properties of tooth enamel
Source: Springerplus. 2013 Oct 2;2:499. doi: 10.1186/2193-1801-2-499 (PMC3795877; doi:10.1186/2193-1801-2-499)
Supplement: Supplementary file 1 — Additional file 1: Table S1: The frequencies and concentrations of 19 trace elements found in 38 human tooth enamel samples analyzed in this study. Table S2. The simple linear regression among trace elements in tooth enamel. The numbers in the table represent the regression coefficient and the color demonstrates the magnitude of the correlation (see legend). Table S3. Simple linear regression between elements and tooth properties. (DOCX 47 KB) [file 40064_2013_569_MOESM1_ESM.docx]

**Supporting Information**

**Table S1.** The frequencies and concentrations of 19 trace elements found in 38 human tooth enamel samples analyzed in this study

|  | N | Minimum (wt/wt) | Maximum (wt/wt) | Mean (wt/wt) | Std. Deviation (wt/wt) |
| --- | --- | --- | --- | --- | --- |
| Al | 38 | .00000 | 2.49811 | .26710 | .56831 |
| B | 38 | .00000 | 1.38873 | .08179 | .30886 |
| Co | 38 | .00000 | .00755 | .00025 | .00124 |
| Cr | 38 | .00000 | .00556 | .00195 | .00128 |
| Cu | 38 | .00000 | .13135 | .03263 | .02897 |
| Fe | 38 | .00000 | .11094 | .02648 | .02640 |
| K | 38 | .00000 | 1.21900 | .43453 | .30241 |
| Mg | 38 | .32198 | 17.36000 | 4.24281 | 3.66623 |
| Mn | 38 | .00000 | .00903 | .00337 | .00311 |
| Mo | 38 | .00000 | .00866 | .00140 | .00191 |
| Na | 38 | .51160 | 33.64000 | 11.17482 | 8.375390 |
| Ni | 38 | .00000 | .53124 | .04814 | .09660 |
| Pb | 38 | .00000 | .09040 | .00650 | .01476 |
| S | 38 | .30590 | 19.54000 | 7.11154 | 4.76416 |
| Sb | 38 | .00000 | .01766 | .00046 | .00286 |
| Se | 38 | .00000 | .13953 | .02203 | .03159 |
| Si | 38 | .00000 | 1.28888 | .11189 | .28714 |
| Ti | 38 | .00000 | .01674 | .00490 | .00500 |
| Zn | 38 | .00940 | 2.63161 | .47965 | .47250 |

**Table S2.** The simple linear regression among trace elements in tooth enamel. The numbers in the table represent the regression coefficient and the color demonstrates the magnitude of the correlation (see legend).

| **Element** | | **Al** | **B** | | **Co** | | **Cr** | | | **Cu** | | **Fe** | | **K** | | **Mg** | | **Mn** | | | **Mo** | | **Na** | | **Ni** | | **Pb** | | | **S** | | **Sb** | | **Se** | | **Si** | | **Ti** | **Zn** |
| --- | --- | --- | --- | --- | --- | --- | --- | --- | --- | --- | --- | --- | --- | --- | --- | --- | --- | --- | --- | --- | --- | --- | --- | --- | --- | --- | --- | --- | --- | --- | --- | --- | --- | --- | --- | --- | --- | --- | --- |
| **Al** | |  | **1.707** | | **-32.18** | | **110.09** | | | **15.73** | | **1.450** | | **-0.32** | | **-0.02** | | **-3.46** | | | **-31.88** | | **-0.008** | | **0.310** | | **-0.517** | | | **-0.01** | | **119.778** | | **0.283** | | **1.823** | | **20.239** | **-0.149** |
| **B** | |  |  | | **-13.87** | | **58.357** | | | **8.789** | | **-0.05** | | **-0.21** | | **-0.01** | | **-9.862** | | | **-5.993** | | **-0.006** | | **-0.05** | | **-0.161** | | | **-0.01** | | **73.732** | | **0.467** | | **1.015** | | **8.595** | **-0.099** |
| **Co** | |  |  | |  | | **0.438** | | | **0.000** | | **-0.007** | | **0.000** | | **0.000** | | **0.054** | | | **-0.015** | | **0.000** | | **-0.001** | | **-0.007** | | | **0.000** | | **-0.015** | | **-0.003** | | **0.000** | | **-0.042** | **0.000** |
| **Cr** | |  |  | |  | |  | | | **0.016** | | **0.003** | | **0.000** | | **0.000** | | **0.095** | | | **0.128** | | **0.000** | | **0.005** | | **-0.013** | | | **0.000** | | **0.067** | | **0.002** | | **0.002** | | **-0.041** | **0.000** |
| **Cu** | |  |  | |  | |  | | |  | | **-0.061** | | **-0.018** | | **-0.001** | | **0.638** | | | **-0.867** | | **-0.001** | | **-0.005** | | **0.024** | | | **-0.001** | | **5.741** | | **0.344** | | **0.087** | | **0.430** | **-0.012** |
| **Fe** | |  |  | |  | |  | | |  | |  | | **0.051** | | **0.002** | | **0.246** | | | **3.834** | | **0.002** | | **0.17** | | **0.180** | | | **0.002** | | **1.384** | | **0.083** | | **0.002** | | **2.39** | **0.029** |
| **K** | |  |  | |  | |  | | |  | |  | |  | | **0.067** | | **24.654** | | | **55.195** | | **0.034** | | **1.317** | | **10.399** | | | **0.057** | | **-25.272** | | **1.788** | | **-0.187** | | **11.015** | **0.401** |
| **Mg** | |  |  | |  | |  | | |  | |  | |  | |  | | **355.7** | | | **95.127** | | **0.396** | | **5.712** | | **166.054** | | | **0.677** | | **-228.03** | | **17.75** | | **-1.33** | | **22.394** | **3.169** |
| **Mn** | |  |  | |  | |  | | |  | |  | |  | |  | |  | | | **0.247** | | **0.000** | | **0.010** | | **0.010** | | | **0** | | **0.014** | | **0.020** | | **0.000** | | **-0.037** | **0.002** |
| **Mo** | |  |  | |  | |  | | |  | |  | |  | |  | |  | | |  | | **0.000** | | **0.01** | | **-0.016** | | | **0.000** | | **-0.082** | | **0.015** | | **-0.001** | | **-0.003** | **0.000** |
| **Na** | |  |  | |  | |  | | |  | |  | |  | |  | |  | | |  | |  | | **33.29** | | **297.46** | | | **1.68** | | **-587.15** | | **38.99** | | **-3.58** | | **272.52** | **10.20** |
| **Ni** | |  |  | |  | |  | | |  | |  | |  | |  | |  | | |  | |  | |  | | **-0.852** | | | **0.009** | | **-0.146** | | **-0.03** | | **0.023** | | **-0.018** | **0.059** |
| **Pb** | |  |  | |  | |  | | |  | |  | |  | |  | |  | | |  | |  | |  | |  | | | **0.002** | | **-0.378** | | **-0.04** | | **0.001** | | **-0.064** | **0.008** |
| **S** | |  |  | |  | |  | | |  | |  | |  | |  | |  | | |  | |  | |  | |  | | |  | | **-368.12** | | **16.94** | | **-1.57** | | **-27.1** | **5.064** |
| **Sb** | |  |  | |  | |  | | |  | |  | |  | |  | |  | | |  | |  | |  | |  | | |  | |  | | **0.008** | | **0.006** | | **0.054** | **-0.001** |
| **Se** | |  |  | |  | |  | | |  | |  | |  | |  | |  | | |  | |  | |  | |  | | |  | |  | |  | | **0.002** | | **1.421** | **0.001** |
| **Si** | |  |  | |  | |  | | |  | |  | |  | |  | |  | | |  | |  | |  | |  | | |  | |  | |  | |  | | **5.722** | **-0.08** |
| **Ti** | |  |  | |  | |  | | |  | |  | |  | |  | |  | | |  | |  | |  | |  | | |  | |  | |  | |  | |  | **0.004** |
| **Zn** | |  |  | |  | |  | | |  | |  | |  | |  | |  | | |  | |  | |  | |  | | |  | |  | |  | |  | |  |  |
|  |  | | |  | |  | |  |  | |  | |  | |  | |  | |  |  | |  | |  | |  | |  |  | |  | |  | |  | |  |  |  |
|  | **0.8<R<1** | | |  | |  | |  |  | |  | |  | |  | |  | |  |  | |  | |  | |  | |  |  | |  | |  | |  | |  |  |  |
|  | **0.6<R<0.8** | | |  | |  | |  |  | |  | |  | |  | |  | |  |  | |  | |  | |  | |  |  | |  | |  | |  | |  |  |  |
|  | **0.4<R<0.6** | | |  | |  | |  |  | |  | |  | |  | |  | |  |  | |  | |  | |  | |  |  | |  | |  | |  | |  |  |  |
|  | **0.2<R<0.4** | | |  | |  | |  |  | |  | |  | |  | |  | |  |  | |  | |  | |  | |  |  | |  | |  | |  | |  |  |  |
|  | **0<R<0.2** | | |  | |  | |  |  | |  | |  | |  | |  | |  |  | |  | |  | |  | |  |  | |  | |  | |  | |  |  |  |

**Table S3.** Simple linear regression between elements and tooth properties

|  | Lattice parameter along a-axis | | | Lattice parameter along c-axis | | | Amide | | | Carbonate type A | | | Carbonate type B | | |
| --- | --- | --- | --- | --- | --- | --- | --- | --- | --- | --- | --- | --- | --- | --- | --- |
| Elements | R | B | Sig | R | B | Sig | R | B | sig | R | B | sig | R | B | sig |
| **Al** | 0.09 | 0.003 | 0.61 | 0.03 | 0 | 0.88 | 0.11 | -0.01 | 0.56 | 0.07 | 0.00 | 0.70 | 0.06 | -0.01 | 0.73 |
| **B** | 0.20 | 0.01 | 0.27 | 0.03 | 0.00 | 0.85 | 0.10 | -0.01 | 0.59 | 0.14 | 0.01 | 0.45 | 0.08 | - 0.02 | 0.66 |
| **Co** | 0.22 | -3.44 | 0.21 | 0.19 | -3.24 | 0.29 | 0.12 | -3.86 | 0.53 | 0.05 | 1.02 | 0.81 | 0.37 | -18.44 | 0.007 |
| **Cr** | 0.20 | -3.33 | 0.26 | 0.38 | -6.97 | 0.03 | 0.07 | 2.11 | 0.72 | 0.28 | 6.49 | 0.12 | 0.12 | 5.57 | 0.53 |
| **Cu** | 0.35 | 0.24 | 0.045 | 0.18 | 0.14 | 0.32 | 0.01 | -0.02 | 0.94 | 0.15 | 0.15 | 0.40 | 0.13 | -0.28 | 0.49 |
| **Fe** | 0.17 | -0.13 | 0.34 | 0.14 | -0.11 | 0.45 | 0.16 | -0.32 | 0.37 | 0.41 | -0.61 | 0.02 | 0.27 | 0.78 | 0.14 |
| **K** | 0.05 | 0 | 0.78 | 0.02 | 0.00 | 0.89 | 0.10 | -0.02 | 0.59 | 0.27 | -0.03 | 0.14 | 0.24 | 0.06 | 0.18 |
| **Mg** | 0.16 | 0.00 | 0.38 | 0.17 | 0.00 | 0.34 | 0.01 | 0 | 0.97 | 0.08 | 0 | 0.65 | 0.18 | 0.004 | 0.32 |
| **Mn** | 0.06 | 0.38 | 0.74 | 0.09 | -0.66 | 0.61 | 0.12 | 1.76 | 0.50 | 0.32 | 3.12 | 0.08 | 0.06 | 1.28 | 0.75 |
| **Mo** | 0.01 | 0.07 | 0.97 | 0.09 | -1.05 | 0.61 | 0.07 | -1.42 | 0.72 | 0.05 | 1.04 | 0.77 | 0.32 | 10.44 | 0.08 |
| **Na** | 0.00 | 0 | 0.98 | 0.04 | 0 | 0.84 | 0.06 | 0 | 0.75 | 0.16 | 0 | 0.38 | 0.32 | 0.00 | 0.07 |
| **Ni** | 0.27 | -0.06 | 0.13 | 0.40 | -0.09 | 0.02 | 0.01 | 0.004 | 0.97 | 0.07 | 0.05 | 0.71 | 0.70 | 0.47 | 0 |
| **Pb** | 0.21 | 0.90 | 0.23 | 0.28 | 1.28 | 0.12 | 0.02 | -0.04 | 0.94 | 0.05 | -0.10 | 0.77 | 0.06 | -0.26 | 0.73 |
| **S** | 0.02 | 0 | 0.93 | 0.07 | 0 | 0.72 | 0.11 | 0 | 0.55 | 0.15 | 0 | 0.42 | 0.43 | 0.01 | 0.014 |
| **Sb** | 0.26 | 1.75 | 0.14 | 0.06 | 0.43 | 0.75 | 0.15 | -2.10 | 0.42 | 0.09 | 0.86 | 0.63 | 0.13 | -2.71 | 0.49 |
| **Se** | 0.59 | 0.36 | 0 | 0.52 | 0.35 | 0.002 | 0.11 | -0.14 | 0.55 | 0.13 | -0.11 | 0.48 | 0.14 | -0.27 | 0.45 |
| **Si** | 0.16 | 0.01 | 0.38 | 0.01 | 0.00 | 0.95 | 0.05 | 0.01 | 0.79 | 0.23 | 0.02 | 0.22 | 0.04 | -0.01 | 0.81 |
| **Ti** | 0.08 | 0.30 | 0.68 | 0.15 | 0.66 | 0.42 | 0.02 | - 0.19 | 0.91 | 0.04 | -0.25 | 0.83 | 0.09 | -1.22 | 0.63 |
| **Zn** | 0.24 | -0.01 | 0.18 | 0.18 | -0.01 | 0.32 | 0.12 | 0.01 | 0.52 | 0.12 | -0.01 | 0.51 | 0.14 | 0.02 | 0.45 |

|  | Crystal size along a-axis | | | Crystal size along c-axis | | | Hardness | | | Lightness | | | Crack length | | |
| --- | --- | --- | --- | --- | --- | --- | --- | --- | --- | --- | --- | --- | --- | --- | --- |
| Elements | R | B | sig | R | B | Sig | R | B | Sig | R | B | Sig | R | B | Sig |
| **Al** | 0.25 | 1.00 | 0.16 | 0.27 | -0.85 | 0.12 | 0.27 | 0.18 | 0.11 | 0.21 | 3.19 | 0.22 | 0.67 | -18.08 | 0.01 |
| **B** | 0.32 | 2.36 | 0.07 | 0.31 | -1.48 | 0.08 | 0.29 | 0.37 | 0.08 | 0.18 | 5.21 | 0.27 | 0.08 | -12.94 | 0.76 |
| **Co** | 0.01 | 25.56 | 0.94 | 0.12 | 172.68 | 0.50 | 0.20 | -63.18 | 0.23 | 0.12 | -887.28 | 0.46 | 0.02 | -252.7 | 0.95 |
| **Cr** | 0.05 | 109.18 | 0.76 | 0.23 | 342.06 | 0.21 | 0.03 | -10.05 | 0.86 | 0.10 | -677.08 | 0.56 | 0.05 | -164.8 | 0.85 |
| **Cu** | 0.40 | 32.87 | 0.02 | 0.16 | -10.07 | 0.37 | 0.21 | 2.94 | 0.20 | 0.18 | 53.83 | 0.29 | 0.20 | -55.94 | 0.47 |
| **Fe** | 0.12 | -10.42 | 0.52 | 0.04 | -2.50 | 0.84 | 0.03 | -0.37 | 0.88 | 0.46 | 153.60 | 0.004 | 0.11 | -15.24 | 0.69 |
| **K** | 0.19 | -1.67 | 0.30 | 0.09 | -0.64 | 0.60 | 0.19 | -0.25 | 0.26 | 0.27 | 7.73 | 0.11 | 0.12 | -1.56 | 0.67 |
| **Mg** | 0.18 | 0.14 | 0.31 | 0.14 | -0.09 | 0.43 | 0.07 | -0.01 | 0.67 | 0.18 | 0.44 | 0.28 | 0.26 | -0.44 | 0.40 |
| **Mn** | 0.21 | 163.78 | 0.23 | 0.34 | 196.81 | 0.05 | 0.28 | -35.01 | 0.10 | 0.18 | -519.68 | 0.27 | 0.44 | -559.4 | 0.09 |
| **Mo** | 0.21 | -261.4 | 0.23 | 0.31 | 288.52 | 0.08 | 0.23 | -46.48 | 0.18 | 0.16 | 749.78 | 0.33 | 0.33 | 607.04 | 0.21 |
| **Na** | 0.001 | 0 | 0.99 | 0.12 | -0.03 | 0.52 | 0.08 | -0.004 | 0.62 | 0.19 | 0.21 | 0.24 | 0.09 | -0.05 | 0.75 |
| **Ni** | 0.30 | -7.46 | 0.08 | 0.21 | 3.99 | 0.23 | 0.11 | -0.45 | 0.52 | 0.08 | 7.48 | 0.63 | 0.09 | 2.65 | 0.75 |
| **Pb** | 0.03 | -13.69 | 0.88 | 0.52 | -199.1 | 0.002 | 0.16 | -4.09 | 0.36 | 0.15 | 91.74 | 0.36 | 0.19 | -142.8 | 0.48 |
| **S** | 0.06 | -0.03 | 0.76 | 0.02 | 0.01 | 0.90 | 0.11 | -0.01 | 0.50 | 0.09 | 0.17 | 0.58 | 0.07 | -0.07 | 0.79 |
| **Sb** | 0.51 | 410.11 | 0.06 | 0.09 | -55.44 | 0.61 | 0.20 | 27.64 | 0.23 | 0.10 | -317.33 | 0.54 | - | - | - |
| **Se** | 0.25 | 18.66 | 0.15 | 0.06 | -3.34 | 0.74 | 0.23 | 2.76 | 0.18 | 0.16 | 44.94 | 0.34 | 0.31 | -115.3 | 0.24 |
| **Si** | 0.28 | 2.39 | 0.10 | 0.32 | -2.03 | 0.07 | 0.30 | 0.42 | 0.07 | 0.15 | 4.65 | 0.36 | 0.19 | -5.45 | 0.49 |
| **Ti** | 0.11 | 52.99 | 0.54 | 0.51 | -192.1 | 0.002 | 0.34 | 27.00 | 0.04 | 0.47 | 832.79 | 0.003 | 0.06 | -50.70 | 0.83 |
| **Zn** | 0.14 | -0.67 | 0.45 | 0.21 | -0.79 | 0.24 | 0.07 | -0.05 | 0.70 | 0.23 | 4.29 | 0.17 | 0.28 | -1.85 | 0.29 |

R: the correlation coefficient; B: the regression coefficient; sig: the significance of Pearson correlation.
